# Claudin18.2 in Advanced Gastric Cancer

**DOI:** 10.3390/cancers15245742

**Published:** 2023-12-07

**Authors:** Rin Inamoto, Naoki Takahashi, Yasuhide Yamada

**Affiliations:** 1 Department of Gastroenterology, Saitama Cancer Center, 780 Komuro, Ina-machi, Kitaadachi-gun, Saitama 362-0806, Japan; inamoto.rin@saitama-pho.jp; 2 Department of Oncology, Comprehensive Cancer Center, National Center for Global Health and Medicine, 1-21-1 Toyama Shinjuku-ku, Tokyo 162-8655, Japan; yayamada@hosp.ncgm.go.jp

**Keywords:** gastric cancer, gastroesophageal junction adenocarcinoma, Claudin18.2, zolbetuximab, chimeric IgG1 monoclonal antibody, ADC, CAR-T

## Abstract

**Simple Summary:**

Gastric cancer (GC) is one of the most common and deadly cancers globally, rendering the development of novel therapeutic strategies an urgent priority. Recently, zolbetuximab, which targets and binds to the transmembrane protein claudin 18 isoform 2 (CLDN18.2). has emerged as a new target therapy in the treatment of advanced GC (AGC). In this review, the molecular and immunological profile of CLDN18.2 in AGC, the results of recent clinical trials, and ongoing clinical trials for patients with CLDN18.2 expressed AGC, and solid tumors were elucidated.

**Abstract:**

Globally, the fifth most common cancer and the fourth leading cause of cancer-related mortality is gastric cancer (GC). Recent clinical trials on solid tumors enrolled patients who possess druggable genetic alterations, protein expression, and immune characteristics. In gastric or gastroesophageal junction (GEJ) cancers, trastuzumab combined with first-line chemotherapy in human epidermal growth factor receptor 2 (HER2)-positive patients and ramucirumab combined with second-line paclitaxel remarkably prolonged overall survival (OS) compared with chemotherapy alone, according to phase 3 trial results. Recently, immune checkpoint inhibitor (ICI) monotherapy was approved as third- or later-line treatment. Chemotherapy plus ICIs as first-line treatment exhibited improved survival compared with chemotherapy alone in HER2-negative patients according to Checkmate 649 trial results. Conversely, systemic chemotherapy prognosis remains poor. although some patients may achieve durable response to treatment and prolonged survival in advanced GC. Recently, a first-in-class, chimeric immunoglobulin G1 monoclonal antibody (zolbetuximab) that targets and binds to claudin 18 isoform 2 (CLDN18.2) has emerged as a new target therapy in GC treatment. Global phase Ⅲ trials revealed that the addition of zolbetuximab to first-line chemotherapy prolonged OS in CLDN18.2-positive and HER2-negative GC patients. This review summarizes recent clinical trials of CLDN18.2-targeted therapy.

## 1. Introduction

Gastric cancer (GC) is the fifth most common cancer and the fourth cause of cancer-related mortality globally [1]. For a long time, the mainstay of treatment for advanced unresectable or metastatic GC is conventional cytotoxic agents [2]. However, with the advent of molecular-targeted therapies, trastuzumab, a human epidermal growth factor receptor 2 (HER2) targeting monoclonal antibody (mAb), became the first-line treatment for HER2-positive GC [3]; moreover, as second-line treatment, ramucirumab, a vascular endothelial growth factor receptor 2-targeting mAb, also showed survival benefit when combined with paclitaxel [4]. Nivolumab, a monoclonal anti-programmed cell death-1 (PD-1) antibody, was approved for third-line or subsequent treatment [5] and subsequently became available as first-line treatment in HER2-negative patients combined with chemotherapy in Japan [6,7]. However, patient prognosis with unresectable or metastatic advanced GC remains unsatisfactory.

**Figure 1:**
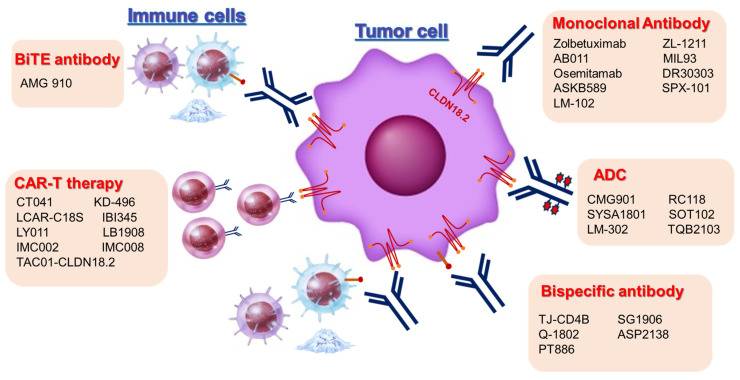
Development of Claudin 18.2-targeted therapy in advanced gastric cancer. Abbreviations: ADC = Antibody drug conjugates; CAR = Chimeric antigen receptor; BiTE = Bispecific T-cell engager.

Zolbetuximab, known as IMAB362, is a mAb that targets and binds to the transmembrane protein claudin 18 isoform 2 (CLDN18.2). Recent phase 3 trials revealed that chemotherapy with zolbetuximab considerably prolonged overall survival (OS) compared with chemotherapy as first-line chemotherapy in HER2-negative patients with CLDN18.2 expression of unresectable GC or GEJ adenocarcinoma [8,9]. In this review, the molecular profiling of CLDN18.2 in advanced GC (AGC), recent clinical trial results, and ongoing clinical trials for patients with CLDN18.2 expressed in AGC and solid tumors are summarized. The overview of recent development of CLDN18.2-targeted therapy in AGC are shown as Figure 1.

## 2. CLND Function in Normal and Tumor Tissues

CLDNs are required for epithelial cell adhesion in vivo and are essential for barrier function [10]. CLDNs are a family of at least 27 transmembrane proteins [10,11,12]. It is believed that the emergence of different types of multiple claudins in different tissues may provide organ-specific barrier functions [13,14]. The distribution of the CLDN family in each organ is shown in Table 1.

CLDNs play roles in signal transduction and tumorigenesis, such as inflammation, growth, and survival of tumor cells, tumor proliferation, epithelial–mesenchymal transition (EMT), metastasis, and cancer stem cells in tumor tissues. For instance, a previous report indicated that CLDN2, CLDN7, and CLDN9 were associated with the promotion of invasion and migration of GC cells [15]. CLDN6 was associated with the promotion of EMT through the Large tumor suppressor kinase 1/2 (LATS1/2)-YAP1(Yes-associated protein 1) pathway in GC tissues [16].

CLDN18 is mainly expressed in the lungs and stomach [17]. Selective CLDN18 gene splicing produces two main variants, CLDN18.1 and CLDN18.2 [18]. CLDN18.1 is highly expressed in the lung alveolar epithelium, expressed at low levels in the epithelial cells of the airways, and is not expressed in the lung endothelial junctions [19]. Additionally, CLDN18.1 maintains alveolar barrier function homeostasis [20,21]. CLDN18.2 protein is a molecule incorporated in the tight junction protein expressed in normal and malignant gastric mucosal epithelial cells, discovered by Tsukita S in 1998 [22]. Tight junctions provide one type of intercellular adhesion and play essential roles in luminal barrier function against solute diffusion through cell gaps, paracellular transport, signal transduction, and the cytoskeleton.

## 3. CLDN18.2 Expression in Normal and Tumor Tissues

In normal gastric mucosal epithelium, CLDN18.2 expression is strictly confined to the differentiated epithelial cells of the gastric mucosa, except for the gastric stem cell zone [23]. However, in malignant tumors, such as gastric, GEJ, esophageal, colon, pancreatic, liver, breast, ovarian, and non-small cell lung tumors, it is also commonly expressed [22,23,24]. In normal tissues, the CLDN18.2 epitope within the tissue adhesion complex is completely inaccessible; however, on malignant transformation, cell adhesion is disrupted, and the CLDN18.2 epitope is exposed on the gastric and GEJ (G/GEJ) adenocarcinoma cell surface, making it a promising target [23].

CLDN18.2-positive GC is more common in signet-ring cell carcinoma and diffuse GC, which correspond to the genomically stable (GS) type of The Cancer Genome Atlas Network Research [25,26,27]. GS-type tumors lean toward a diffuse pathologic subtype and occur at a relatively young age [28].

For each clinical trial in patients with GC, the positive criteria for CLDN18.2 differ. The cutoff for immunohistochemistry (IHC) staining for CLDN18.2 positivity was defined as moderate-to-strong CLDN18.2 expression (CLDN18.2 expression ≥2+ in ≥40% of tumor cells) in the FAST trial [29]. Conversely, the cutoff for IHC staining was a strong CLDN18.2 expression (IHC 2+/3+ in ≥75% of tumor cells) in two recent phase III trials (SPOTLIGHT and GLOW) [8,9].

## 4. Prognostic Role of CLDN18.2 in AGC

The prognostic significance of CLDN18.2 in GC varies in different studies, and a consensus remains lacking. Sanada Y et al. reported that CLDN-18 downregulation via a quantitative reverse transcription polymerase chain reaction was observed in 84 of 146 patients with GC and correlated with poor survival (*p* = 0.0346) [30]. Furthermore, Jun et al. evaluated CLDN-3, CLDN-7, and CLDN-18 expression through IHC in 134 patients with AGC who underwent surgery. In patients with CLDN-18 expression, OS was longer than those without CLDN-18 expression (5-year survival rate, 90.5% vs. 64.8%) [31]. Conversely, Wang et al. revealed significant associations between CLDN18.2 expression and OS (hazard ratio [HR] 1.37, *p* = 0.031) [32]. Arnold et al. reported that a high CLDN18.2 expression by IHC was not associated with OS in a large Caucasian esophagogastric or gastric cohort with 414 patients [33]. Dottermusch et al. reported that no significant correlation was found between tumor-specific survival and CLDN18.2 expression in tumor cells in 430 patients with advanced GC (*p* = 0.439) [34].

## 5. Prevalence of CLDN18.2 and Biomarker Overlap in AGC

Currently, HER2, programmed cell death-ligand 1 (PD-L1), and deficient mismatch repair (dMMR)/microsatellite instability (MSI) are first-line biomarkers for GC. Additionally, in advanced G/GEJ adenocarcinoma, clinical trials of fibroblast growth factor receptor 2 (FGFR2)-targeted antibodies are underway, and FGFR2b may also become one of the future treatment targets and biomarkers [35,36]. Previous reports that investigated CLDN18.2-positive GC prevalence, biomarker prevalence, and overlap were summarized (Table 1) [37,38,39,40,41]. There is biomarker overlap in CLDN18.2-positive patients, and the majority of CLDN18.2-positive tumors were HER2 negative, proficient mismatch repair/microsatellite stable, Epstein–Barr virus (EBV) negative, and had a PD-L1 combined positive score (CPS) <1 [37] as shown in Table 2. In clinical practice, the treatment strategy for patients with biomarker overlap is not established, and further development of combination or sequential treatment of these molecular targets are warranted in the future.

## 6. Clinical Trials of Anti-CLDN18.2 Agent for AGC or Solid Tumors

Promising therapeutics targeting CLDN18.2 for GC, mAbs, bispecific antibodies (BsAbs), chimeric antigen receptor (CAR)-T cell therapy redirected to target CLDN18.2, and antibody–drug conjugates (ADCs) have been developed for cancer immunotherapy. Regarding CLDN18.2 clinical trials, as of August 2023, there are 43 ongoing trials highlighting advanced unresectable GC on the Clinical Trials.gov (NCT) website, including three phase II, 12 phase I/II, and 28 phase I clinical trials. For the agent types, thirteen trials employed mAbs, six trials employed BsAbs, twelve trials employed CAR-T cell therapy, nine trials employed ADCs, and three trials applied others. In this review article, we elucidate the recent progress in the advances in the novel CLDN18.2-targeted agents (Table 3).

### 6.1. mAbs

#### 6.1.1. Zolbetuximab (IMAB 362)

Zolbetuximab is an immunoglobulin G1 (IgG1) chimeric mAb that specifically binds to CLDN18.2 [42]. For the preclinical zolbetuximab data, Mitnacht-Kraus et al. reported the characteristics and mechanisms of IMAB362 in vitro using CLDN18.2-expressing cell lines and in mouse tumor xenografts [43]. Moreover, the antitumor activity of zolbetuximab was evaluated in human GC cell lines and xenografts in mice treated using chemotherapy with or without zolbetuximab. In both in vivo and in vitro, zolbetuximab was highly selective for CLDN18.2. Zolbetuximab mediated effective and target-selective antibody-dependent cellular cytotoxicity (ADCC) against GC cell lines with endogenous CLDN18.2 expression and induced complement-dependent cytotoxicity (CDC)-mediated lysis of CLDN18.2-expressing tumor cells.

In patients with advanced GC and GEJ cancer, a phase I dose-escalation study of zolbetuximab monotherapy was conducted [44]. Twenty-nine patients were screened, and fifteen patients met the inclusion criteria, were sequentially enrolled into five dose groups (33 to 1000 mg/m^2^), and received IMAB362 monotherapy. As dose-limiting toxicity (DLT) did not occur within a 4-week treatment in any of the dose groups, a maximum tolerated dose was not established. Overall, 13 out of 15 patients experienced any adverse event (AE), and the most common treatment-related AEs (TRAEs) were gastrointestinal disorders. Gastrointestinal toxicities (nausea and vomiting) were observed immediately after initiation of infusion. Although a phase I study is neither designed nor powered to evaluate antitumor activity, most patients (n = 12/15; 80%) had progressive disease after IMAB 362 intravenous infusion. According to the pharmacokinetics (PK) and pharmacodynamics (PD) data, a dose of 300–600 mg/m^2^ every 2 weeks was selected.

The MONO trial is a multicenter, open-label, phase IIa study assessing the efficacy and safety of zolbetuximab as a single agent in patients with metastatic or advanced gastric/gastroesophageal junction/esophageal adenocarcinoma [45]. Patients manifesting with moderate or strong (2+/3+) CLDN18.2 membrane staining intensities in ≥50% of tumor cells were included. The objective response rate (ORR) and disease control rate (DCR) were 9% (n = 4) and 23% (n = 10), respectively. Of the patients who achieved clinical benefit, 90% (n = 9/10) had moderate-to-high CLDN18.2 expression in ≥70% of tumor cells. TRAEs occurred in 81.5% (n = 44/54) of patients; the most common were nausea (61%), vomiting (50%), and fatigue (22%). The common grade 3 or 4 TRAEs were nausea (15%) and vomiting (22%).

The ILUSTRO trial is phase II non-randomized study assessing zolbetuximab efficacy (800 mg/m^2^ loading dose and then at 600 mg/m^2^ every 3 weeks thereafter) in patients with advanced/metastatic G/GEJ adenocarcinoma whose tumors have high (≥75% of tumor cells) or intermediate (≥50% but <75% expression of tumor cells) CLDN18.2 expression [46]. This trial comprised four cohorts. Cohort 1A enrolled 30 patients with high CLDN18.2 expression to receive third-line or zolbetuximab monotherapy later. The ORR and DCR were 0% and 44.4%, respectively. The median progression-free survival (PFS) and OS were 1.54 (95% confidence interval [CI]: 1.31–2.56) and 5.62 months (95% CI: 2.27–11.53), respectively. Any-grade AEs were nausea (63.3%), abdominal pain (40.0%), vomiting (36.7%), asthenia (26.7%), decreased appetite (23.3%), anemia (20.0%), and pyrexia (20.0%). Cohort 2 included 20 patients with high CLDN18.2 expression to receive mFOLFOX plus zolbetuximab as a first-line treatment. The DCR and ORR were 100% and 71.4%, respectively. The median PFS was 17.8 months (95% CI: 8.05–25.69). Any-grade AEs included nausea (90.5%); vomiting (66.7%); decreased neutrophil count and appetite (42.9% each); neutropenia, diarrhea, and fatigue (38.1% each); abdominal pain and peripheral sensory neuropathy (33.3% each); constipation and pyrexia (28.6% each); and anemia, decreased weight, back pain, and myalgia (23.8%). Cohort 3A enrolled only three patients to receive third-line or later zolbetuximab plus pembrolizumab. No response was observed, and the DCR was 66.7%. Nausea, constipation, pyrexia, and decreased appetite (N = 2 each) were the most frequently reported. Moreover, cohort 4A (safety cohort) and 4B (N = 50) are conducted for evaluating efficacy and safety of combination of zolbetuximab, mFOLFOX6, and nivolumab as first-line treatment in G/GEJ cancer (NCT03505320). In cohort 4A, patients will receive a loading dose of 800 mg/m^2^ of zolbetuximab with 240 mg of nivolumab and mFOLFOX6 on cycle 1 day 1, followed by 400 mg/m^2^ of zolbetuximab with 240 mg of nivolumab and mFOLFOX6 every 2 weeks. Tolerability and safety of the zolbetuximab, mFOLFOX6, and nivolumab combination will be evaluated during a 2-week DLT assessment period. Cohort 4B will receive this combination at the dose level determined in cohort 4A. This study is now ongoing, and results are anticipated.

The FAST trial is a randomized phase II trial of EOX plus zolbetuximab versus EOX alone as first-line treatment for advanced CLDN18.2-positive GC and gastroesophageal cancer [29]. Patients with moderate (2+) or strong (3+) CLDN18.2 expression in at least 40% of tumor cells were enrolled. To assess CLDN18.2 expression in patients with GC, CLDN18.2 expression was evaluated at a central laboratory using the CLAUDETECT™ 18.2 histology kit. Among 686 patients, 334 (48.7%) patients were diagnosed as CLDN18.2 positive. The EOX plus zolbetuximab group exhibited significantly longer PFS (7.5 vs. 5.3 months, HR 0.44, 95% CI: 0.29–0.67, *p* < 0.0005) and OS (13 vs. 8.3 months, HR 0.55, 95% CI: 0.39–0.77, *p* < 0.0005). The ORR with EOX with zolbetuximab was 39%, compared with 25% with EOX alone. In patients with CLDN18.2 expression in ≥70% of tumor cells, a significant benefit of PFS (HR 0.38) and OS (HR 0.44) were observed. Conversely, in patients with CLDN18.2 expression in 40–69% of tumor cells, PFS (4.3 vs. 4.1 months, HR 0.71, 95% CI: 0.32–1.57, *p* = 0.497) and OS (8.3 vs. 7.4 months, HR 0.78, 95% CI: 0.40–1.49, *p* = 0.401) were not significantly different. Based on these results, the eligibility criteria in the phase 3 trial (SPOTLIGHT and GLOW trials) were CLDN18.2-positive patients defined as ≥75% of tumor cells with moderate-to-strong CLDN18 staining.

The SPOTLIGHT trial is a multicenter, randomized, double-blind, phase III trial designed to evaluate zolbetuximab plus mFOLFOX6 superiority over standard chemotherapy (mFOLFOX6) in the primary treatment of CLDN18.2-positive, HER2-negative gastric and GEJ adenocarcinomas with unresectable locally advanced or metastatic disease [8]. Patients with moderate (2+) or strong (3+) CLDN18.2 expression in ≥75% of tumor cells were included. Approximately 38% of patients who were evaluable for CLDN18.2 were CLDN18.2-positive. PD-L1 CPS of 5 or more was observed in 41 (13%) of the 311 assessed patients. Chemotherapy consisted of zolbetuximab (800 mg/m^2^ as the initial loading dose, 600 mg/m^2^ every 3 weeks thereafter) plus mFOLFOX6 (every 2 weeks) or placebo plus mFOLFOX6. The mFOLFOX6 plus zolbetuximab group demonstrated a significantly longer median PFS (10.6 vs. 8.7 months, HR 0.750, 95% CI: 0.60–0.94, *p* = 0.0066) and median OS (18.2 vs. 15.5 months, HR 0.55, 95% CI: 0.60–0.94, *p* = 0.0053). The response rates were similar in the zolbetuximab (60.7%) and the placebo group (62.1%). The median DoR was 8.51 months in the zolbetuximab group. The placebo group was comparable at 8.11 months; however, the third quartile was longer in the zolbetuximab (29.9 months) and the placebo group (15.5 months). The frequency of grade 3 or worse AEs in the zolbetuximab and placebo groups was 242 (87%) and 216 (78%), respectively. The most common AEs during the zolbetuximab group dosing were nausea (82% and 61% in the zolbetuximab and the placebo group, respectively), vomiting (67% and 36%), and decreased appetite (47% and 33%). Notably, patients who underwent total gastrectomy had a lower frequency of severe gastrointestinal AEs than those who did not.

The GLOW trial was a multicenter, randomized phase III trial to evaluate the efficacy of zolbetuximab on CAPOX in HER2-negative and CLDN18.2-positive patients with metastatic or advanced G/GEJ adenocarcinomas [9]. Similar to the SPOTLIGHT trial, patients with moderate (IHC 2+) or strong (IHC 3+) CLDN18.2 expression in at least 75% of the tumor cells were enrolled. PD-L1 CPS ≥5 was noted in 225 (78.1%) of the 288 assessed patients. Chemotherapy consisted of zolbetuximab (intravenous infusion of zolbetuximab, 800 mg/m^2^ as initial loading dose, 600 mg/m^2^ every 3 weeks thereafter) plus CAPOX or placebo plus CAPOX. PFS and OS were remarkably prolonged with zolbetuximab added to CAPOX (median PFS: 8.2 vs. 6.8 months, HR 0.69, 95% CI: 0.54–0.87, *p* = 0.0007, median OS: 14.4 vs. 12.2 months, HR 0.77, 95% CI: 0.62–0.97, *p* = 0.0118). The ORR was 42.5% (95% CI, 36.4–48.9) in the zolbetuximab group vs. 40.3% (95% CI, 34.2–46.6) in the placebo group, and DoR was 6.1 (95% CI, 5.0–8.1) vs. 6.08 months (95% CI, 4.4–6.3), respectively, with no significant additive effect. AEs of grade ≥3 occurred in 72.8% in the zolbetuximab group vs. 69.9% in the placebo group.

The results of clinical trials of treatment including zolbetuzimab are summarized in Table 4.

#### 6.1.2. AB011

AB011 is a humanized anti-CLDN18.2 IgG1 mAb, and preclinical studies have demonstrated potential synergistic effects between AB011 and cytotoxic drugs. A phase I study of AB011 monotherapy (part 1) and CAPOX with AB011 (part2) in patients with advanced solid tumors was conducted (NCT04400383) [47]. Part 1 cohort included patients with CLDN18.2-positive, advanced G/GEJ adenocarcinomas and pancreatic cancer. Dose levels of 1 to 30 mg/kg of AB011 were evaluated by using a 3 + 3 design with dose escalation and dose-escalation steps, and 20 mg/kg and 30 mg/kg were further assessed. Part 2 cohort included patients both with advanced G/GEJ adenocarcinomas and CLDN18.2 expression (IHC 2+/3+ ≥ 40%). Fourteen patients were treated with AB011 at 1 to 30 mg/kg in the dose-escalation step, and 21 patients in the dose-expansion step in part 1. In the 30 mg/kg group, one DLT was grade 3 dyspnea. Among 20 patients with measurable disease, the DCR was 60% (N = 12), and a patient with GC (30 mg/kg) was assessed as complete response (CR). In part 2, no patients experienced DLT or TRAEs associated with treatment discontinuation or death. Among 23 patients, the DCR and ORR were 100% and 65.2%, respectively.

#### 6.1.3. Osemitamab (TST001)

TST001 is a novel humanized anti-CLDN18.2 IgG1 mAb with enhanced Fc binding affinity to FcγRIIIa (CD16a) and ADCC activity. In preclinical studies, TST001 upregulated PD-L1 expression on low-to-medium CLDN18.2-positive tumor cells and demonstrated stronger ADCC, CDC, and antibody-dependent cellular phagocytosis (ADCP) activity than zolbetuximab [48].

A TST001 multi-cohort phase Ⅰ/Ⅱ trial combined with CAPOX subsequently reported an encouraging 100% ORR in 15 patients as first-line treatment of patients with CLDN18.2-expressing G/GEJ adenocarcinoma; 11 patients (73.3%) showed a partial response (PR), and 4 (26.7%) had a stable disease (SD). The treatment was well tolerated with mostly grade 1/2-related TRAEs, such as nausea (59%), vomiting (39%), and increased ALT (18%). PFS and DoR for this cohort are ongoing (NCT04495296) [49]. The preclinical data showed that the antitumor efficacy of TST001 combined with an anti-PD-1 antibody and chemotherapy was more superior than the anti-PD-1 antibody with chemotherapy or combination of TST001 with chemotherapy [48]. Thus, TST001 in combination with the anti-PD-1 antibody nivolumab is being investigated in a phase Ⅰ/Ⅱ trial as a first-line treatment for metastatic gastric and GEJ adenocarcinoma (NCT04495296) [50].

#### 6.1.4. ASKB589

ASKB589 is a humanized anti-CLDN18.2 IgG1 mAb with high affinity and enhanced ADCC. Preliminary results of phase I/II, dose-escalation, and expansion study of ASKB589 for patients with advanced solid tumors were reported (NCT04632108) [51]. In this study, the maximum tolerated dose (MTD), safety and tolerability, PK, PD, and efficacy of ASKB589 monotherapy (part A) and in combination with chemotherapy (part B) were evaluated. In part A, patients received ASKB589 intravenously at doses of 0.3 to 20 mg/kg every 3 weeks. In part B, patients with G/GEJ cancers received ASKB589 at doses of 3 to 15 mg/kg in combination with CAPOX. Among 51 patients who received ASKB589, no DLTs were observed. In expansion cohort, patients were enrolled into 6, 10, and 15 mg/kg doses. In part A, 17 and 13 patients were enrolled in escalation cohort and expansion cohort, respectively. The most common AEs were nausea (53%), vomiting (43%), hypoalbuminemia (40%), and decreased appetite (30%). For the nine evaluable patients (≥10 mg/kg ASKB589), five patients (45%) had decreased tumor size, whereas two patients achieved a PR. Furthermore, six patients had SD for a DCR of 89%. In part B, 17 patients (81%) had TRAEs, including nausea (76%), vomiting (66%), hypoalbuminemia (52%), granulocytopenia (38%), and hypoleukemia (33%). For the 12 evaluable patients (≥6 mg/kg ASKB589), all had decreased tumor size, and nine patients achieved PR for an ORR of 75%, and DCR was 100%. ASKB589 demonstrated a manageable safety profile of up to 20 mg/kg doses and promising antitumor activity.

#### 6.1.5. LM-102

LM-102 is a recombinant humanized mAb targeting CLDN18.2. Enrollment of a phase I, first-in-human, dose-escalation study to evaluate the safety and tolerability and antitumor activity of LM-102 monotherapy in patients with CLDN18.2-positive advanced solid tumors (NCT04735796) was initiated last May 2021. In the dose-escalation cohorts, the 3 + 3 escalation design was used. Dose escalation consists of five ascending dose levels (3 to 40 mg/kg). This study was already discontinued.

#### 6.1.6. ZL-1211

ZL-1211 is a humanized IgG1 mAb targeting CLDN18.2. Site mutations (S239D and I332E) were introduced at the Fc portion of ZL-1211 to enhance ADCC, ADCP, and CDC. The preclinical findings conducted by Konno et al. revealed that ZL-1211 can target not only CLDN18.2 high-expressing tumor cells but also intermediate/low-expressing tumor cells, suggesting that ZL-1211 may have a better clinical response than zolbetuximab [52,53]. In the phase Ⅰ/Ⅱ trial with ZL-1211 plus CAPOX, 19 patients with CLDN18.2-positive advanced solid tumors were enrolled in the dose-escalation phase to receive ZL-1211 at 1 mg/kg (n = 3), 5 mg/kg (n = 7), 10 mg/kg (n = 4), and 20 mg/kg (n = 5) (NCT05065710) [54]. No DLTs were observed as of the cutoff date. The most common TRAEs were gastrointestinal disorders, including nausea (57.9%), vomiting (31.6%), and abdominal pain (15.8%). TRAEs led to treatment interruption in five patients (26.3%) and treatment withdrawal in one patient (5.3%). SD as the best overall response was shown in six patients (66.7%), among nine patients whose antitumor activity was evaluable. Three patients demonstrated tumor regression: 24.9% in a patient with GC in the 10 mg/kg cohort.

#### 6.1.7. MIL93

MIL93 is a humanized anti-CLDN18.2 IgG1 mAb. A multicenter, dose-escalation and expansion phase 1 study of MIL93 for patients with advanced solid tumors was initiated from January 2021 in China (NCT04671875). In the dose-escalation cohort, six dose levels (0.3 to 30 mg/kg, every 3 weeks) of MIL93 were evaluated. In this study, accelerated titration was adopted for the first two dose levels, and the 3 + 3 design was used. In the dose-expansion phase, patients with CLDN18.2-positive cancers received the selected RP2D. The preliminary result of the MIL93 monotherapy (n = 30) elucidated that MIL93 was well tolerated (0.3–30 mg/kg), and DLT was observed in one patient during the 3-week window at 30 mg/kg [55]. However, there were no DLTs in three additional patients at 30 mg/kg, and MTD has not been achieved. The most common TRAEs were nausea (60%), hypoalbuminemia (46.7%), vomiting (43.3%), anemia (36.7%), hyponatremia (33.3%), hypocalcemia (23.3%), appetite loss (23.3%), asthenia (23.3%), and increased aspartate aminotransferase (20.0%). Among the 25 patients, two patients with GC achieved PR and DCR of 44%.

#### 6.1.8. DR30303

Compared to IgG antibodies, Variable domain of Heavy chain of Heavy chain (VHH) antibodies have many advantages, including lower costs for production (antibodies that bind strongly to target antigens can be rapidly developed by antibody library methods), higher productivity (antibodies can be produced in large quantities at low cost using *E. coli* or other bacteria), and greater stability (heat resistance, denaturing agents, surfactants, pH). DR30303 is a humanized anti-CLDN18.2 heavy chain antibody Fc fusion protein (VHH-Fc) and demonstrates a highly selective to CLDN18.2 [56]. Anti-tumor activity of DR30303 monotherapy was observed in various gastric and pancreatic cell-derived xenograft (CDX) mouse models with a minimum effective dose (0.3 mg/kg), which led to TGI ≥ 90%. DR30303 showed strong synergistic effects with paclitaxel, gemcitabine, epirubicin, oxaliplatin, and fluorouracil in patient-derived xenograft (PDX) mouse models and the CDX model. Recently, an open, phase I, dose-escalation and dose-expansion study to evaluate the safety, tolerability, PK, and preliminary efficacy of DR30303 in patients with advanced solid tumors was initiated, and enrollment is ongoing (NCT05639153).

#### 6.1.9. SPX-101

SPX-101 is a humanized anti-CLDN18.2 antibody. Preclinical data showed high affinity and specificity, high effector cell-mediated cytotoxicity, low immunogenicity, and in vivo tumor control efficacy in xenograft [57]. Recently, a phase 1, open-label study to evaluate SPX-101′s safety, tolerability, PK, and efficacy in patients with advanced or refractory solid tumors is now ongoing (NCT05231733).

### 6.2. BsAbs/Bispecific T-Cell Engager (BiTE) Antibody

#### 6.2.1. TJ-CD4B (ABL111)

TJ-CD4B (ABL111) is a novel CLDN18.2 dependent 4-1BB BsAb. It binds to 4-1BB and CLDN18.2 and activates 4-1BB signaling to enhance T-cell activation in a CLDN18.2-dependent manner. 4-1BB is expressed in activated T-cells, including CD8 T-cells, CD4 T-cells, regulatory T cell (Treg), natural killer (NK) T-cells, cytokine-activated NK cells, activated dendritic cells (DCs), eosinophils, mast cells, and, intriguingly, endothelial cells in some metastatic tumors [58], and is a co-stimulatory receptor that stimulates multiple immune cell function. The preclinical TJ-CD4B in vitro and in vivo animal experiments exhibited a strong tumor growth inhibition of CLDN18.2-expressing tumor cells. TJ-CD4B also increased tumor-infiltrating lymphocytes, whereas it showed no impact on peripheral lymphocytes [59]. TJ-CD4B is in a phase I clinical trial as a treatment for CLDN18.2-positive patients with advanced or metastatic solid tumors, including patients with gastric and GEJ, esophageal, and pancreatic ductal adenocarcinoma (NCT04900818). The primary endpoints include DLTs, AE incidence and severity, and MTD of TJ-CD4B.

#### 6.2.2. AMG 910

AMG 910 is a BiTE antibody construct designed to engage CD3-positive T-cells with CLDN18.2-positive G/GEJ adenocarcinoma cells. Phase I study to evaluate the efficacy and safety of AMG 910 is now ongoing in patients with CLDN18.2-positive G/GEJ cancer (NCT04260191) [60]. Patient enrollment is open in North America, Europe, and Asia. The key inclusion criteria were aged ≥ 18 years, histologically diagnosed as metastatic or locally advanced unresectable CLDN18.2-positive G/GEJ adenocarcinoma. The dose-exploration phase (n ≤ 34) will assess the MTD and RP2D according to safety, efficacy, and PD data prior to reaching an MTD.

#### 6.2.3. Q-1802

Q-1802 is a humanized bispecific antibody that targets both CLDN18.2 and PD-L1. A phase 1a/1b, multicenter, dose-escalation, and dose-expansion study of Q-1802 monotherapy are now ongoing in patients with advanced solid tumors who refractory to standard treatment (NCT04856150). In the dose-escalation phase, an accelerated titration followed by a 3 + 3 design was used to assess safety and tolerability of Q-1802 (dose range 0.1–20 mg/kg) and determine the MTD. The interim study results (N = 29) were reported, and no DLTs up to 20 mg/kg of Q-1802 were observed. Two dose groups, 10 and 20 mg/kg, were extended in the phase 1b study [61]. The most common treatment-related AEs were gastrointestinal AEs (89.7%), including nausea (62.1%), vomiting (62.1%), abdominal pain (27.6%), and gastroesophageal reflux disease (24.1%). irAEs transpired in seven participants, including abnormal thyroid function, fatigue, rash, and arthritis. Among the nine patients who had measurable lesions in the dose-expansion cohort, two patients achieved PR, and four achieved SD as the best overall response.

#### 6.2.4. SG1906

SG1906 is a recombinant anti-CLDN18.2/CD47 IgG1 bispecific antibody, developed by Sumgen for the treatment of patients with locally advanced unresectable or metastatic solid tumors. In China, a phase Ia/Ib study to evaluate the safety, tolerability, and efficacy of SG1906 in patients with CLDN18.2-positive advanced solid tumors is now ongoing (NCT05857332). In the phase Ia part, an accelerated titration–Bayesian optimal interval (AT-BOIN) design is used with seven dose cohorts (0.1 to 12 mg/kg), administered every 2 weeks. The dose-expansion part (phase Ib) includes patients with histologically diagnosed as CLDN18.2-positive advanced G/GEJ cancer or pancreatic cancer who were refractory to standard treatment.

#### 6.2.5. ASP2138

ASP2138 is an asymmetric 2 + 1 format, T-cell BsAb with bivalent humanized anti-CLDN18.2 antigen-binding fragments and a monovalent anti-CD3 single-chain variable fragment. ASP2138 showed anti-tumor activity against CLDN18.2-expressing gastric or pancreatic cancer cells [62]. The antitumor activities were dependent on the effector cell to target cell ratio and CLDN18.2-positve cells. Phase I/Ib study of ASP2138 is now ongoing in patients with CLDN18.2-positive gastric or GEJ adenocarcinoma or pancreatic adenocarcinoma (NCT05365581).

#### 6.2.6. PT886

PT886 is a novel BsAb that targets CLDN18.2 and CD47. PT886 utilizes IgG1 to enhance ADCC by NK cells and ADCP by macrophages. An open-label, phase I study of PT886 is ongoing in patients with advanced G/GEJ adenocarcinoma and pancreatic cancer, for which a standard treatment was failed (NCT02178241). The study comprised dose-escalation cohort and dose expansion cohort. Patients will receive PT886 as monotherapy (0.1 to 6 mg/kg, weekly).

### 6.3. Peptide Fused to CLDN 18.2 Antibody

#### LB4330

LB4330, as a novel peptide fused to CLDN18.2 antibodies, targets the tumor antigen associated CD8+ T cells. LB4330 shows high affinity to human anti-CLDN18.2 (14 pM) and CD8+T cells. This drug activates CD8 T cells in the tumor microenvironment and has potential alone or in combination with PD-1/PD-L1 mAb for the treatment of advanced solid tumors, especially for CLDN18.2-positive pancreatic ductal adenocarcinoma. A phase I trial of LB4330 in patients with advanced solid tumors is currently ongoing (NCT05707676) [63].

### 6.4. ADCs

#### 6.4.1. CMG901

CMG901 is a CLDN18.2 ADC, which has showed preclinical antitumor activity through monomethyl auristatin E (MMAE)-mediated cytotoxicity through bystander killing, ADCC, and CDC. The result of phase Ia dose-escalation study of CMG901 in patients with refractory solid tumors was recently reported (NCT04805307) [64]. This trial uses a modified 3 + 3 dose-escalation design to evaluate the safety and tolerability in patients with advanced G/GEJ, pancreatic cancers, and other solid tumors. CMG901 was administered at doses of 0.3 to 3.4 mg/kg. Grade ≥ 3 TRAEs were observed in 3/27 (11%) patients. MTD was not attained. In CLDN18.2-positive G/GEJ cancer patients (N = 8), ORR and DCR were 75.0% and 100%, respectively. The recruitment for phase 1b dose-expansion trial in gastric/GEJ adenocarcinoma, pancreatic cancer, and other solid tumors is currently ongoing.

#### 6.4.2. SYSA1801

SYSA1801 is a CLDN18.2-targeted ADC delivering MMAE, a microtubule inhibitor. SYSA1801 revealed a considerable antitumor activity in vitro and in vivo in multiple cell lines and PDXs in gastric, pancreatic, and lung cancer expressing CLDN18.2 in preclinical studies [65]. Of the 33 patients with resistant recurrent solid tumors included in the phase I trial, 26 (78.8%) had GC. In part I, patients received up to 3 mg/kg of SYSA1801. In the 17 evaluable GC patients, the ORR was 47.1% (PR in eight patients), and the DCR was 64.7% (SD in three patients). TRAEs of any grade occurred in 25 patients (75.8%), of which eight (24.2%) were grade 3/4. Nausea (42.4%) and vomiting (36.4%) were the most common TRAEs (occurring in >20% of patients). Grade 3 nausea and vomiting as DLTs occurred at the 3 mg/kg dose. The optimal dose of SYSA1801 is being explored (NCT05009966) [66].

#### 6.4.3. CPO102

CPO102 is an anti-CLDN18.2 mAb conjugated to MMAE conjugates. MMAE (Vedotin) is an antimitotic agent approximately 100–1000-fold more potent than doxorubicin that acts through tubulin polymerization blockade. Phase I of a CPO102 monotherapy (dose-escalation and dose-expansion) study is now ongoing in patients with advanced pancreatic or G/GEJ cancers (NCT05043987). Part A will explore once-every-3-weeks (Q3W) dosing per a standard 3 + 3 dose-escalation design. Upon attaining an RP2D, part B will commence in two groups, in approximately 15 patients with advanced pancreatic cancer and 15 patients with advanced G/GEJ cancers.

#### 6.4.4. LM-302

LM-302 (TPX-4589), a novel ADC developed to target CLDN18.2, comprised a recombinant humanized anti-CLDN18.2 IgG1 mAb (LM-102) coupled with cytotoxic payload MMAE. As preclinical data, in both high- and low-expressing gastric and pancreatic CLDN18.2 tumor models, LM-302 potently inhibited tumor cell proliferation in vitro and reduced tumor growth in vivo. Furthermore, LM-302 demonstrated superior internalization and efficacy compared with zolbetuximab in a GC tumor model [67]. Phase I study of a LM-302 in patients with CLDN18.2-positive advanced solid tumors is now under evaluation (NCT05001516). Moreover, a phase I/II, open-label, multicenter study to evaluate the safety and tolerability of LM-302 plus toripalimab (anti-PD-1 antibody) in patients with advanced solid tumors is now underway (NCT05188664).

#### 6.4.5. RC118

RC118 is a recent advanced ADC used to treat patients with solid tumors positive for CLDN18.2 expression. A phase I/IIa study of RC118 for patients with locally advanced solid tumors with positive CLDN18.2 expression is now ongoing (NCT05205850). In the phase I dose-escalation phase, seven dose groups will be preset (0.25 to 3.0 mg/kg). In the phase IIa cohort expansion phase, participants were grouped according to different tumor types, with 30 participants in each group, initially considering the inclusion of patients with CLDN18.2-positive GC, esophageal, GEJ, and pancreatic cancer.

#### 6.4.6. SOT102

SOT102 is a novel CLDN18.2-targeting antibody–drug conjugate based on a proprietary monoclonal antibody conjugated to a PNU159682 (topoisomerase inhibitor) derivative via site-specific sortase-mediated conjugation. In numerous patient-derived xenograft models such as gastric, pancreatic, liver, colon, and lung adenocarcinomas, the single-agent therapeutic SOT102 activity was shown. In all CLDN18.2-positive models, CRs were noted, irrespective of staining intensity. SOT102 remains stable without any significant payload loss in both in vitro and animal models [68]. A multicenter phase I/II trial is conducted to evaluate the safety and efficacy of SOT102 as monotherapy and in combination with standard of care treatment in patients with gastric and pancreatic adenocarcinoma (CLAUDIO-01, NCT05525286). This trial will evaluate the MTD and RP2D of SOT102 administered as monotherapy (part A) and in combination with first-line chemotherapy (mFOLFOX6 with nivolumab and nab-paclitaxel/gemcitabine; part B) and SOT102 efficacy administered as monotherapy (part C) and in combination with first-line chemotherapy (part D) in patients with advanced inoperable or metastatic gastric and GEJ adenocarcinoma or inoperable or metastatic pancreatic adenocarcinoma.

#### 6.4.7. TQB2103

TQB2103 is a CLDN18.2-targeting ADC independently developed by Sino Biopharm. Upon binding to tumor cells expressing CLDN18.2 on the surface, TQB2103 is internalized and transported to the lysosome, where the cytotoxic payload is released, thereby selectively killing the tumor cell through deoxyribonucleic acid (DNA) damage. Through a bystander effect, it can kill adjacent negative tumor cells. A phase I trial evaluating tolerance, safety, and efficacy of TQB2103 for injection in patients with advanced cancers is now underway (NCT05867563).

### 6.5. CAR-T Cell Therapy

#### 6.5.1. CAR-T Cell (CT041)

CT041, a CAR-T cell therapy for CLDN18.2, involves genetically engineered autologous T-cells expressing a CAR targeting CLDN18.2. Preclinical data show that CT041 is effective in CLDN18.2-positive GC cell lines and patient-derived tumor xenograft models [69]. In China, interim analysis results of an ongoing phase 1 study of CT041 in patients with CLDN18.2-positive gastrointestinal cancers (NCT03874897) were published [70]. In this interim analysis, the first 37 patients were included, including gastric and GEJ cancer (N = 28), pancreatic cancer (n = 5), and other gastrointestinal tumor types (n = 4). According to discussions with the data safety monitoring committee, the 2.5 × 10^8^ CAR-T cell dose was recommended for the dose-expansion phase. The most common AEs of ≥grade 3 were preconditioning-related hematologic toxicities in 37 (100%), leukopenia in 31 of 37 (83.8%), neutropenia in 25 of 37 (67.6%), anemia in 15 of 37 (40.5%), and thrombocytopenia in 6 of 37 (16.2%) patients. No grade 3 or higher cytokine release syndrome (CRS) was noted. Thirty (83.3%) patients demonstrated tumor regression.

A phase Ⅰb, open-label, multicenter study in the U.S., a clinical trial of CT041 CAR-T cells in patients with advanced gastric or other specified gastrointestinal cancers has demonstrated acceptable safety and promising antitumor efficacy (NCT04404595) [71]. Eleven patients (five gastric and six pancreatic cancers) were treated using CT041 at a dose between 2.5 and 4 × 10^8^ cells. Among five GC patients, three patients were evaluable for response by the data cutoff date. One patient achieved CR, and two patients attained PR. CAR-T cell expansion correlated with circulating tumor DNA reduction. The median DoR and the PFS had not been reached. DLTs, TRAEs, ≥grade 3 CRS, immune effector cell-associated neurologic syndrome, or severe gastrointestinal-related AEs were not noted.

As for other CAR-T cell therapies, an open-label, single-arm, clinical trial of CT048 in patients with advanced solid tumors is now ongoing in China (NCT05393986).

#### 6.5.2. LCAR-C18S

LCAR-C18S was developed as a CAR T-cell therapy targeting CLDN18.2 by Nanjing Legend Biotech Co. This phase I, single-arm, open-label, dose-finding, and expansion study to evaluate the safety, tolerability, and antitumor efficacy profiles of cell-based LCAR-C18S in patients with CLDN18.2-positive advanced solid tumors (NCT04467853) has been terminated.

#### 6.5.3. LY011

LY011 is an anti-CLDN18.2 third-generation CAR T-cell therapy prepared from allogeneic T-lymphocytes that have been genetically modified to target TAA CLDN18.2 through lentivirus vector technology with potential immuno-stimulating and antineoplastic activities. A phase I study of LY011 for CLDN18.2-positive patients with advanced GC and pancreatic cancer is now ongoing (NCT04977193 and NCT04966143). As of 31 January 2021, these studies have completed the evaluation of DLT and efficacy after the first treatment of four patients with advanced solid tumors (N = 3: GC; N = 1: pancreatic cancer). Among these patients, two patients achieved PR, and two patients achieved SD. The ORR was 50.0% (N = 2/4), and the DCR was 100% (N = 4/4). In the lowest-dose group of 1 × 10^6^ CAR-T cells/kg, the ORR was 66.67% (2/3), and the DCR was 100% (3/3). No serious AEs or AEs leading to participant dropout have been identified. Three of four patients experienced grade 1 CRS after CAR-T cell reinfusion [72].

#### 6.5.4. IMC002

IMC002 is an autologous CAR-T cell therapy based on an anti-CLDN18.2 VHH antibody with high specificity, with no cross-reactivity to CLDN18.2. A phase 1, open-label, multicenter, dose-escalation study is conducted to evaluate safety, feasibility, and preliminary efficacy of IMC002 in patients with CLDN18.2-positive gastrointestinal tumors, including, but not limited to, advanced GC, esophagogastric junction adenocarcinoma, and advanced pancreatic cancer (NCT05946226). Approximately 9–18 patients with CLDN18.2-positive advanced gastrointestinal tumors will be sequentially enrolled into three dose-escalation cohorts to evaluate the safety and feasibility of autologous IMC002 treatment. Patients will undergo leukapheresis and IMC002 product preparation following enrolment. If the disease progresses promptly, patients may receive bridging therapies as determined by the investigator. After treatment with cyclophosphamide, fludarabine, and nab-paclitaxel lymphodepletion, patients will be assigned to one of three dose-escalation cohorts: 1.0 × 10^8^, 2.5 × 10^8^, or 5.0 × 10^8^ CAR-T cells.

#### 6.5.5. IMC008

IMC008 is an activating receptor natural killer group 2, member D (NKG2D) receptor-modified autologous CAR-T cells targeting CLDN18.2. A phase I, open-label, multicenter, dose-escalating study to evaluate the safety and preliminary efficacy of IMC008 in the treatment of CLDN18.2-positive solid tumors is now underway (NCT05837299).

#### 6.5.6. KD-496

KD-496, a novel bispecific CAR-T cell, which simultaneously recognizes NKG2D ligands and CLDN18.2, revealed superior antitumor efficacy and safety in vitro and in vivo. KD-496 CAR-T cells potently respond to GC and possess a more efficient tumor elimination than single CAR, such as KD-025 and KD-182 CAR-T cells in a PDX model with a good safety profile [73]. A phase 1, single-arm, single-center, open-label study to evaluate the safety and efficacy of KD-496 CAR-T cell infusion in the treatment of advanced NKG2DL/CLDN18.2-positve solid tumors is now ongoing (NCT05583201).

#### 6.5.7. IBI345

IBI345 is the first universal “modular” CAR-T cell product developed by Innovent based on Roche’s proprietary technology. It is an internationally pioneered highly differentiated CAR-T cell product with a new mechanism of action. IBI345 comprises anti-CLDN18.2 antibodies and “modular” CAR-T cells, where the anti-CLDN18.2 antibody recognizes the tumor antigen, thus calibrating and amplifying the antigen signal, and guides “modular” CAR-T cells to enter the tumor to initiate the cytotoxic and antitumor activity of CAR-T cells [74].

#### 6.5.8. LB1908

LB1908 is an autologous CAR-T therapy selectively targeting CLDN18.2 via a high-affinity VHH antibody. A phase 1, multicenter study is conducted to evaluate CLDN18.2-targeting CAR-T cells (LB1908) in patients with advanced gastric, GEJ, esophageal, or pancreatic adenocarcinoma (NCT05539430).

#### 6.5.9. TAC01-CLDN18.2

The T-cell antigen coupler (TAC) is a chimeric receptor that facilitates the re-direction of T-cells to tumor cells and activates T-cells by co-opting the endogenous T-cell receptor complex, eliciting safe and durable antitumor responses. CLDN18.2-TAC T-cells demonstrated a specific anti-CLDN18.2 response, as no cytotoxicity or increases in proliferation or activation were observed when they were co-cultured with CLDN18.2-negative target cells. The in vitro repeat killing assay demonstrated strong and persistent antitumor activities of CLDN18.2-TAC T-cells against CLDN18.2-expressing target cells [75]. A phase 1 and 2 study investigating the safety and efficacy of autologous TAC T-cells in patients with unresectable, locally advanced, or metastatic CLDN18.2-positive solid tumors is now underway (NCT05862324).

### 6.6. mRNA-Based Therapy

Messenger RNA (mRNA) therapies are gaining attention not only in infectious disease vaccines but also in cancer immunotherapies. mRNA can be produced within hours at a relatively low cost by in vitro transcription (IVT). Furthermore, unlike DNA therapeutics, mRNA is not integrated into the genome and is therefore safe without the risk of insertional mutagenesis [76]. mRNA-based drug development was conceived by Wolff et al. in 1990. They injected mRNA encoding various proteins into mouse muscles and successfully expressed these proteins [77]. An mRNA is inherently structurally unstable and can be degraded by ribonuclease (RNase) or trapped by endosomes prior to reaching its site of action [78]. Thus, lipid nanoparticles (LNPs) were developed as vehicles to protect and deliver RNA [79].

#### BNT141

BNT141 is a novel RNA-based therapeutic agent consisting of mRNA encoding a mAb against CLDN18.2 encapsulated in LNP. BNT141 is absorbed by hepatocytes when intravenously administered and binds to the cellular plasma membrane, releasing mRNA into the cell. The mRNA is then translated by ribosomes to produce the anti-CLDN18.2 mAbs, which are released into the blood. The antibody specifically targets, binds to, and inhibits CLDN18.2 expressed in tumor cells. This may kill CLDN18.2-expressing tumor cells. A phase I/II trial of BNT141 is ongoing to evaluate the safety and efficacy of BNT141 in patients with CLDN18.2-positive solid tumors, including G/GEJ adenocarcinomas (NCT04683939). This trial comprises three cohorts: (1) BNT141 monotherapy, (2) BNT141 in combination with nab-paclitaxel and gemcitabine, and (3) dose expansion [80].

## 7. Conclusions

CLDN18.2 is a novel biomarker, and zolbetuximab in combination with oxaliplatin-based chemotherapy regimens was proven effective in two phase III trials (SPOTLIGHT and GLOW trials). Moreover, combination therapy with ICI and novel treatment approaches such as ADC, BsAbs, BiTE, CAR T-cell therapy, and mRNA-based therapy are under development with the expectation of therapeutic efficacy. Additionally, establishing optimal treatment strategies is a future challenge, when other biomarkers such as HER2, PD-L1 CPS, and MSI status overlap. In the future, further development of CLDN18.2-targeted agents leading to an improved prognosis in patients with unresectable or advanced recurrent metastatic gastric and GEJ adenocarcinoma is anticipated.

## Figures and Tables

**Table 1:** The distribution of the claudin family in each organ.

Tissue Distribution	Claudin Family
Skin	claudin1, claudin2, claudin3, claudin4, claudin6, claudin8, claudin12, claudin17, claudin20, claudin23
Lung	claudin1, claudin3, claudin4, claudin5, claudin18
Stomach	claudin 18
Liver	claudin1, claudin2, claudin3, claudin5
Brain	claudin1, claudin5
Kidney	claudin1, claudin2, claudin3, claudin4, claudin7, claudin8, claudin10, claudin13, claudin15, claudin16, claudin19
Cornea	claudin1, claudin2, claudin3, claudin7, claudin14
Inner ear	claudin8, claudin9, claudin10, claudin12, claudin14, claudin18
Breast	claudin4, claudin7
Pancreas	claudin2, claudin3
Prostate	claudin4
Small intestine	claudin10, claudin15
Duodenum	claudin20, claudin22
Colon	claudin15
Taste receptor cells	claudin17, claudin23
Trachea	claudin22
Placenta	claudin23
Schwann’s cell	claudin19

**Table 2:** Summary of biomarker overlap with CLDN 18.2 in advanced gastric cancer.

	Retrospective Study, Single Center Cohort			Clinical Trials of CLDN 18.2 Antibody		
CLDN18.2+, IHC	≥40% Tumor Cells	≥75% Tumor Cells	≥75% Tumor Cells	≥75% Tumor Cells	≥75% Tumor Cells	≥75% Tumor Cells
CLDN18.2+ (%)	52	33	24	38	38	39
References	Jia K et al. [36]	Pelino A et al. [37]	Kubota Y et al. [38]	SPOTLIGHT Trial [8]	GLOW Trial [9]	SPOTLIGH/GLOW [39]
HER2+ (%)	21	15	15	-	-	-
dMMR (%)	14	13	5	-	-	-
PD-L1 CPS < 1 (%)	21	74	26	-	-	-
PD-L1 CPS ≥ 5 (%)	-	18	42	13	22	17
EBV+ (%)	19	6	4	-	-	-
Diffuse type (%)	29	40	48	29	34	49
Intestinal type (%)	38	46	52	25	14	39
Mixed/Other (%)	33	12	-	46	51	-

Abbreviations: IHC = immunohistochemistry; CLDN = claudin; HER2 = human epidermal growth factor receptor 2; dMMR = mismatch repair deficient; PD-L1; programmed cell death 1-ligand 1; CPS = combined positive score; EBV = Epstein–Barr virus.

**Table 3:** Summary of ongoing clinical trials conducted in the therapeutic agents targeting CLDN18.2 in advanced gastric cancer.

Type of Drug	Drug	Clinical Trial Number	Phase	Subjects	Primary Endpoint
mAb	Zolbetuximab (IMAB362)	NCT03505320	II	Advanced unresectable GEJ cancer or GC	ORR
mAb	AB011	NCT04400383	I	Solid tumor, GC, PC	DLT, AEs
mAb	TST001	NCT04495296	I	Advanced solid tumors	DLT, AEs, MTD
mAb	ASKB589	NCT04632108	I, II	Advanced solid tumors	DLT, AEs, MTD
mAb	BNT141	NCT04683939	I, II	Advanced solid tumors	TEAE, DLT
mAb	LM-102	NCT04735796	I	Advanced solid tumors	DLT, AEs, MTD
mAb	LM-102	NCT05008445	I, II	Advanced solid tumors	DLT, AEs, MTD, RP2D
mAb	ZL-1211	NCT05065710	I, II	Advanced solid tumors	MTD, TRAE, ORR
mAb	IMC 002	NCT05946226	I	advanced digestive system tumor	DLT
mAb	MIL93	NCT04671875	I	advanced solid tumors	AEs
mAb	DR30303	NCT05639153	I	advanced digestive system tumor	DLT, TEAE, MTD, RP2D
mAb	TST001	NCT04396821	I, II	advanced solid tumors	AEs, MTD, RP2D
mAb	SPX-101	NCT05231733	I	advanced solid tumors	DLT, MTD, MAD
BiTE antibody	AMG 910	NCT04260191	I	GC or GEJ adenocarcinoma	DLT, AEs
BsAbs	Q-1802	NCT04856150	I	Advanced solid tumors	DLT
BsAbs	SG1906	NCT05857332	I	advanced solid tumors	AEs, MTD, MAD, RP2D
BsAbs	ASP2138	NCT05365581	Ⅱ	GC, GEJ cancer, PC	DLT, AEs
BsAbs	PT886	NCT05482893	I	GC, PC	DLT, RP2D, MTD
BsAbs	IBI315	NCT05608785	I, II	GC, GEJ cancer	AEs
Specific bi-functional molecule	LB4330	NCT05707676	I	advanced solid tumors	DLT, AEs, RP2D, MTD
ADC	CMG901	NCT04805307	I	Advanced solid tumors, ST	DLT, TEAE, ORR, RP2D
ADC	SYSA1801	NCT05009966	I	Advanced solid tumors, GC, GEJ cancer, PC	DLT, AEs, RP2D
ADC	CPO102	NCT05043987	I	GC, PC	DLT
ADC	LM-302	NCT05161390	I, II	Advanced solid tumors	DLT, AEs, RP2D, MTD
ADC	LM-302	NCT05161390	I, II	advanced solid tumors	DLT, AEs, RP2D, MTD
ADC	LM-302	NCT05001516	I, II	advanced solid tumors	DLT, AEs
ADC	RC118	NCT04914117	I	advanced solid tumors	DLT, AEs, RP2D, MTD
ADC	SOT102	NCT05525286	I, II	GC, GEJ cancer, PC	AEs, RP2D, ORR
ADC	TQB2103	NCT05867563	I	advanced solid tumors	DLT, MTD, RP2D
ADC+anti PD-1mAb	LM302+JS001	NCT05934331	Ⅱ	GC, PC	PFS
CAR-T cell	CT041	NCT03159819	I	Advanced GC, PC	AEs
CAR-T cell	CT041	NCT03874897	I	Advanced solid tumors	DLT, MTD
CAR-T cell	CT041	NCT04404595	I	GC, PC	AEs, MTD, ORR
CAR-T cell	CT041	NCT04581473	I, II	GC, PC, GEJ adenocarcinoma	AEs, MTD, PFS
CAR-T cell	LCAR-C18S	NCT04467853	I	Advanced solid tumors	DLT, TEAE, RP2D
CAR-T cell	LY011	NCT04977193	I	Advanced gastric adenocarcinoma	AEs, MTD
CAR-T cell	IMC002	NCT05472857	I	Advanced solid tumors	SAE, DLT
CAR-T cell	IMC008	NCT05837299	I	advanced solid tumors	DLT
CAR-T cell	KD-496	NCT05583201	I	NKG2DL+solid tumors	TEAE, DLT
CAR-T cell	IBI345	NCT05199519	I	solid tumors	AEs
CAR-T cell	CT048	NCT05393986	I	advanced solid tumors	DLT, MTD
CAR-T cell	LB1908	NCT05539430	I	GC, GEJ cancer, esophageal cancer, PC	RDE, RP2D
TAC T cell	TAC01-CLDN18.2	NCT05862324	I, II	solid tumors	DLT, AEs

Abbreviations: mAb = monoclonal antibody; BiTE = bispecific T-cell engager; BsAbs = Bispecific antibody; ADC = antibody-drug conjugate; CAR = Chimeric antigen receptor; TAC = T-cell antigen coupler; CLDN = claudin; GEJ = gastro-esophageal junction. GC = gastric cancer; PC = pancreas cancer; ORR = objective response rate; AE = adverse event; DLT = dose limiting toxicity; MTD = maximum tolerated dose; RP2D = recommended phase 2 dose; PFS = progression-free survival; TEAE = treatment emergent adverse event; SAE = serious adverse event; RDE = recommended dose for expansion.

**Table 4:** Summary of the phase II and III trials of chemotherapy with zolbetuximab in advanced gastric or GEJ cancer.

Study Name	MONO	ILUSTRO		FAST	SPOTLIGHT	GLOW
		(Cohort 1A)	(Cohort 2)			
Trial Number	NCT04396821	NCT03505320		NCT01630083	NCT03504397	NCT03653507
References	[43]	[44]		[27]	[8]	[9]
Phase	II	II		II	III	III
Patient number	54	30	21	246	566	507
Treatment line	≥2nd line	≥3rd line	1st line	1st line	1st line	1st line
CLDN18.2-positive	≥50% cells (+)	≥50% cells (+)	≥50% cells (+)	≥40% cells (+)	≥75% cells (+)	≥75% cells (+)
Experimental arm	ZOL	ZOL	mFOLFOX6 + ZOL	EOX + ZOL	mFOLFOX6 + ZOL	CapeOX + ZOL
Control arm	−	−	−	EOX	mFOLFOX	CapeOX
ORR (%)	9	0	71.4	25.0 vs. 39.0	62.1 vs. 60.7	48.8 vs. 53.8
PFS (months)	−	1.54	17.81	5.3 vs. 7.5	8.7 vs. 10.6	6.8 vs. 8.2
				HR 0.4	HR 0.75	HR 0.69
				*p* < 0.0005	*p* = 0.0066	*p* = 0.0007
OS (months)	−	5.62	−	8.3 vs. 13.0	6.8 vs. 8.2	12.2 vs. 14.4
				HR 0.55	HR 0.69	HR 0.77
				*p* < 0.0005	*p* = 0.0007	*p* = 0.0118

Abbreviations: CLDN = claudin; ORR = objective response rate; DCR = disease control rate; PFS = progression-free survival; OS = overall survival; ZOL = zolbetuximab; mFOLFOX = modified FOLFOX (5-FU/Leucovorin/Oxaliplatin); EOX = epirubicin/oxaliplatin/capecitabine; CapeOX = capecitabine/oxaliplatin; vs. = versus; HR = hazard ratio.

## Abbreviations

GC	Gastric cancer
CLDN 18.2	Claudin 18 isoform 2
AGC	Advanced GC
GC	Gastric cancer
GEJ	Gastroesophageal junction
G/GEJ	Gastric/ Gastroesophageal junction
HER2	Epidermal growth factor receptor 2
CLDN	Claudin
OS	Overall survival
LAST 1/2	Large tumor suppressor kinase 1/2
YAP1	Yes-associated protein 1
ICI	Immune checkpoint inhibitor
mAb	Monoclonal antibody
PD-1	Programmed cell death-1
GS	Genomically stable
IHC	Immunohistochemistry
PD-L1	Programmed cell death-ligand 1
dMMR	Deficient mismatch repair
MSI	Microsatellite instability
FGFR2	Fibroblast growth factor receptor 2
EBV	Epstein–Barr virus
CPS	Combined positive score
BsAbs	Bispecific antibodies
CAR	Chimeric antigen receptor
ADC	Antibody–drug conjugates
IgG1	Immunoglobulin G1
ADCC	Antibody-dependent cellular cytotoxicity
CDC	Complement-dependent cytotoxicity
ADCP	Antibody-dependent cellular phagocytosis
DLT	Dose-limiting toxicity
AE	Adverse event
TRAEs	Treatment-related AEs
PK	Pharmacokinetics
PD	Pharmacodynamics
ORR	Objective response rate
DCR	Disease control rate
PFS	Progression-free survival
DoR	Duration of response
mFOLFOX	Modified FOLFOX (5-FU + Leucovorin + Oxaliplatin)
EOX	Epirubicin + Oxaliplatin + Capecitabine
HR	Hazard ratio
CI	Confidence interval
CAPOX	Capecitabine + Oxaliplatin
CR	Complete response
PR	Partial Response
SD	Stable disease
FcγRIIIa	CD16a
mRNA	Messenger ribonucleic acid
RP2D	Recommended phase II dose
MTD	Maximum tolerated dose
VHH	Variable domain of Heavy chain of Heavy chain
CDX	Cell-derived xenograft
PDX	Patient-derived xenograft
NK	Natural killer
DCs	Dendritic cells
BiTE	Bispecific T-cell engager
AT-BOIN	Accelerated titration-Bayesian optimal interval
TAA	Tumor-associated antigen
MMAE	Monomethyl auristatin E
Q3W	Once-every-3-weeks
CRS	Cytokine release syndrome
DNA	Deoxyribonucleic acid
NKD2G	Natural killer group 2, member D
TAC	T-cell antigen coupler
mRNA	Messenger RNA
RNase	Ribonuclease
LNPs	Lipid nanoparticles
